# A Cognitive Behavioral Therapy-Informed Self-Management Program for Acute Respiratory Failure Survivors: A Feasibility Study

**DOI:** 10.3390/jcm10040872

**Published:** 2021-02-20

**Authors:** Megan M. Hosey, Stephen T. Wegener, Caroline Hinkle, Dale M. Needham

**Affiliations:** 1 Department of Physical Medicine and Rehabilitation, Johns Hopkins School of Medicine, Baltimore, MD 21287, USA; swegener@jhmi.edu (S.T.W.); chinkle2@jhmi.edu (C.H.); dale.needham@jhmi.edu (D.M.N.); 2 Department of Medicine, Division of Pulmonary and Critical Care Medicine, Johns Hopkins School of Medicine, Baltimore, MD 21287, USA; 3 Outcomes after Critical Illness and Surgery (OACIS) Research Group, Johns Hopkins University, Baltimore, MD 21287, USA; 4 Department of Psychology, University of Maryland, Baltimore County, Baltimore, MD 21250, USA

**Keywords:** critical care, critical care outcomes, intensive care units, anxiety, cognitive behavior therapies

## Abstract

Background: The number of people surviving critical illness is rising rapidly around the globe. Survivorship comes at a cost, with approximately half of patients with acute respiratory failure (ARF) experiencing clinically significant symptoms of anxiety, and 32–40% of survivors having substantial anxiety symptoms in the months or years after hospitalization. Methods: This feasibility study reports on 11 consecutive ARF patients receiving up to six sessions of a psychological intervention for self-management of anxiety. Results: All 11 patients accepted and received the psychological intervention. Four patients did not fully complete all 6 sessions due to death (*n* = 1, 2 sessions completed), and early hospital discharge (*n* = 3, patients completed 2, 3 and 5 sessions). The median (IQR) score (range: 0–100; minimal clinically important difference: 13) for the Visual Analog Scale-Anxiety (VAS-A) pre-intervention was 70 (57, 75) points. During the intervention, all 11 patients had a decrease in VAS-A, with a median (IQR) decrease of 44 (19, 48) points. Conclusions: This self-management intervention appears acceptable and feasible to implement among ARF patients during and after an ICU stay.

## 1. Introduction

The number of survivors of acute respiratory failure (ARF) requiring mechanical ventilation in an intensive care unit (ICU) continues to rise around the globe [1]. However, survival comes at a “cost,” with survivors frequently experiencing long-lasting impairments in mental health, including depression, anxiety, and Post-Traumatic Stress Disorder (PTSD) [2,3]. To help address these impairments, there have been changes in clinical practice in the ICU. Evidence-based guidelines for ARF patients recommend minimizing sedation to reduce associated side effects (e.g., delirium, prolonged bedrest) [4,5]. Given decreased sedation, evaluating and addressing psychological distress for ARF patients in the ICU is presumably more feasible and important to consider. Notably, approximately 50% of ARF patients experience clinically significant levels of anxiety in the intensive care unit (ICU) [6,7,8,9], including feelings of terror, fear of death, loss of control, and eroded sense of self [7,10,11,12]. Moreover, clinically significant symptoms can be long-lasting after ARF, with 32–40% of survivors having clinically significant anxiety symptoms enduring for months or years [3,13].

Preliminary studies suggest that early non-pharmacological interventions increase self-efficacy and may reduce longer-term mental health symptoms in ICU survivors [14]. First, a pre-post evaluation of a non-specific psychotherapeutic intervention in the ICU was associated with substantially lower anxiety and depression symptoms at 12-month follow-up. However, the psychological interventions used were not defined and cannot be readily replicated in other centers [15]. Second, two case studies suggest that cognitive behavioral therapy (CBT)-informed treatments conducted by a psychologist can expedite liberation from mechanical ventilation [16]. Third, a pilot randomized-controlled trial (RCT) of a CBT-informed protocol, administered by psychologists on a burn unit, reported that survivors (*n* = 50) had significantly reduced anxiety symptoms and post-traumatic stress disorder (PTSD) at 1 month follow-up [17]. Fourth, a national, cluster-randomized RCT conducted in the UK evaluated a 3 session, nurse-implemented, CBT-informed intervention delivered during an ICU stay [8]. This RCT did not demonstrate improved PTSD symptoms at 6-month follow-up; However, this study demonstrated reduced anxiety symptoms for those who received all 3 treatment sessions. Lessons from this RCT suggest: (1) intervention dose may be an important consideration in psychological interventions, and (2) the intervention should be defined, refined, and iterated for efficacy by psychologists and mental health professionals prior to eventual transition to other health professionals [18]. Collectively, these initial studies offer promising preliminary results; however, their feasibility, acceptability, and efficacy is under-studied in ARF patients and in the ICU and hospital setting.

CBT-informed self-management interventions are non-pharmacological treatments that have been demonstrated to reduce anxiety and improve functional outcomes in many medical populations [19,20,21,22], including individuals with pulmonary conditions [23,24,25]. These interventions place patients at the center of their recovery by providing them the knowledge and skills to manage symptoms [26]. Since such interventions are focused on managing specific problems and symptoms, they must be tailored to specific medical populations and settings [18,26]. Once developed and manualized, these interventions can be implemented by trained non-psychologists, such as nurses, physical therapists, and peer supports [27,28,29,30].

Given the need for additional study in this area, we adapted and implemented a CBT- informed self-management protocol for ARF patients. The purpose of this study was to evaluate the feasibility and acceptability of this protocol when used in routine psychology consultation and treatment in the Medical Intensive Care Unit at the Johns Hopkins Hospital, in Baltimore USA.

## 2. Methods Section

This feasibility study included a convenience sample of 11 consecutive patients in a medical intensive care unit who received consultation, assessment and treatment for anxiety using the protocol by a licensed clinical psychologist (MMH; [31]) who was clinically working two days per week during a 6-month period (November 2018–April 2019). To be eligible for the intervention, patients were experiencing ARF with mechanical ventilation and not delirious, as per the Confusion Assessment Method-ICU (CAM-ICU) screening tool and assessment by the psychologist. See Table 1 for full eligibility criteria. Feasibility was defined as >70% of intervention sessions completed, and <15% drop-out (defined as patient request to discontinue) among survivors by hospital discharge. Acceptability was evaluated via patient agreement to initiate with the intervention and continued participation in each subsequent intervention session.

Efficacious, CBT-informed self-management interventions in other medical populations consist of a total of 3 to 10 sessions with common treatment components to provide a patient “toolbox” of disease-related education and coping skills. Self-management in acute respiratory failure (SMARF) is a 6-session intervention which was adapted from existing protocols to improve anxiety in acute respiratory failure survivors [19,32,33]. Specific adaptation was based on previous research and prior clinical feedback from ARF patients, including focus on strategies for managing anxiety during mechanical ventilator liberation, problem solving to obtain education about medical treatment and prognosis, and coping skills related to rehabilitation engagement and uncertain hospital environments. Each session has a consistent structure, including anxiety measurement at the beginning of each session via VAS-A, education about the session topic, application of skills, and suggested “putting it into practice” activities, allowing patients to practice skills between sessions. The SMARF protocol is shown in Table 2.

Patients had initial sessions in the medical intensive care unit (MICU) and were followed in the acute care hospital unit until the 6 sessions were completed. Delirium was assessed at each session and sessions were rescheduled if the patient was delirious. Sessions with intubated patients were conducted using individually tailored communication procedures, including talking tracheostomy devices, handwriting, communication boards, offering multiple choice and yes/no questions, and lip reading, depending on the needs, abilities and preferences of each patient. The interventionist participated in the SPEACS-2 Communication Training Program [34]. Each session lasted approximate 30–40 min. This feasibility study was approved by the Johns Hopkins Institutional Review Board.

## 3. Results

**Figure 1:**
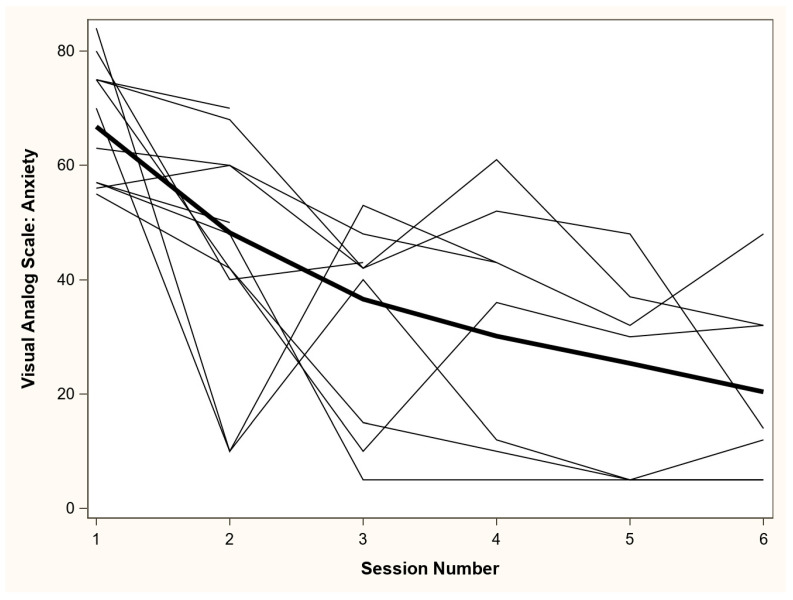
Visual Analog Scale-Anxiety (VAS-A) scores across self-management intervention session. VAS-A scores range from “not anxious at all” (score = 0) to “most anxious I have ever felt” (score = 100). The bold line represents a locally estimated scatterplot smoothing (LOESS) regression line across all data points, and all other lines represent values from each patient across each of the self-management sessions.

The 11 patients were 55% female and 55% White (see Table 3). All 11 (100%) patients accepted to initiate the SMARF intervention, with a goal to deliver the 6 SMARF sessions. Four patients did not complete all 6 sessions due to death (*n* = 1, 2 sessions completed), and hospital discharge (*n* = 3, each patient completed 2, 3 and 5 sessions). All 11 ARF patients experienced delirium prior to the psychology consultation. One patient had a clinical deterioration and recurrence of delirium, forcing cancellation of all subsequent sessions, with eventual patient death. No other patients had episodes of delirium requiring cancellation or rescheduling of sessions. The VAS anxiety score (range: 0 to 100) was high immediately prior to the start of intervention (i.e., “baseline”), with a median (interquartile range [IQR]) score of 70 (57, 75). At the last SMARF session, all 11 patients experienced a decrease from baseline. In all patients, and in the subset of 7 patients who completed all 6 sessions, the median (IQR) decrease in VAS-A from baseline was 44 (21, 48) and 51 (44, 62) points, respectively (Table 4; Figure 1). No patient declined any of 54 SMARF intervention sessions.

## 4. Discussion

This study evaluates the feasibility of a psychologist-delivered, CBT-informed, self-management intervention for patients with ARF, delivered in the ICU and acute care unit as part of routine clinical care [31]. The 11 patients in this feasibility study had similar ratings of anxiety when compared to other cohorts of patients receiving non-pharmacological interventions for anxiety in the ICU [7,8,9]. Feasibility goals of >70% of intervention sessions completed (across all patients), and <15% drop-out of survivors (as defined by patient request to discontinue) by hospital discharge were met. No sessions were declined by patients, suggesting that they found the intervention acceptable. Based on this study, the SMARF intervention is feasible and acceptable to non-delirious patients without history of severe psychiatric or cognitive comorbidities. SMARF also demonstrates potential for improvement in anxiety symptoms. To evaluate the efficacy of the intervention, a randomized controlled trial is needed.

Patients in this case series had long ICU and hospital lengths of stay [8,35]. A longer length of stay may make psychological symptoms more apparent and provide time for consultation. In addition, patients with increased length of stay may be more likely to have problems that trigger psychology consultation [36].

Important considerations regarding feasibility of the intervention include delirium, which is common in ARF patients in the ICU [37]. Inattention, a cardinal feature of delirium, would prevent the necessary learning for patients to benefit from the intervention. Hence, patients only received the intervention once delirium resolved. Despite all patients having delirium prior to start of the intervention, only 1 of the 11 patients experienced delirium after start of the intervention that was associated with clinical deterioration that required cessation of the intervention and subsequent death. Another consideration regarding feasibility was availability of adequate time to conduct the 30–40 min intervention given that critically ill and hospitalized patients often have unpredictable schedules whereby medical interventions (e.g., medications administration, imaging, procedures) may interrupt SMARF sessions. Finally, fatigue is common in critically ill patients hospitalized patients. To help ensure feasibility of the intervention, the following strategies were helpful: (1) communicating regularly with the ICU and ward clinical staff about patient availability/scheduling and maintaining flexibility in scheduling sessions with an expectation that patients may require more than 1 attempt to initiate a session, (2) eliciting the patient’s communication preferences (e.g., writing, lip reading) and (3) providing written materials to assist with recall and practice of skills learned during the session.

In this small case series, anxiety reduction was substantial and exceeded the minimum clinically important difference for the VAS-A instrument [38]. This preliminary observation may be important, as there is great need for effective non-pharmacological strategies for managing anxiety in the ICU and hospital. Despite guidelines suggesting reduced use of benzodiazepines and other sedating medications, hospital and ICU providers have not been provided with evidence or support for helpful alternatives [4]. Medications remain the most common means of reducing anxiety [6]. This can have important harmful effects including increased delirium, worsened anxiety, and lengthened ICU and hospital stays [39,40].

This study has several important limitations. First, the small convenience sample, lack of a control group, and limited information about previous history of mental health symptoms and treatment preclude any definitive statements about the efficacy of the SMARF intervention. A second limitation is that most ICUs do not have access to a psychologist with expertise in care of ARF patients. However, experts (e.g., trained psychologists) must develop successful behavioral intervention protocols. Training and implementation by other healthcare providers or via technology is an important subsequent step after the initial development of an efficacious protocol [18]. Third, it is possible that the natural history of anxiety is a decline over time and that the intervention was not causally related to the observed reduction in VAS-A scores. In the future, it will be beneficial to obtain patient/stakeholder feedback about the appropriateness, timing, and duration of sessions.

## 5. Conclusions

The goal of this small study is to explore the acceptability and feasibility of a psychological intervention implemented in the ICU and acute care hospital unit to lay the foundation for future work evaluating the efficacy, effectiveness, and implementation of an intervention to improve long-term mental health outcomes for ARF survivors. The SMARF intervention, delivered in the ICU and acute care unit, is feasible and acceptable for non-delirious ARF patients. SMARF has the potential to improve self-management of anxiety in hospitalized ARF patients. Additional studies are needed to further evaluate and extend these findings.

## Figures and Tables

**Table 1:** Eligibility Criteria for Patients.

Inclusion Criteria	Exclusion Criteria
≥18 years old English speaking ARF with mechanical ventilation via endotra cheal tube or tracheostomy ^1^ Expected hospital stay of ≥7 days ^1,2^ Alert (e.g., RASS sedation score = −1, 0, or 1) ^1^ Not delirious (e.g., negative CAM-ICU score) ^3^ Presence of clinically significant anxiety symp toms at initial consultation (VAS-A score ≥50) ^4^	Pre-existing cognitive impairment (e.g., dementia) ^1^ History of major psychiatric illness (i.e., psychotic disorder bipolar disorder, suicide attempt in past 24 months, perva sive developmental disorder) ^1^ Anticipated discharge to hospice, primary focus on pallia tive care, or >90% probability of in-hospital death ^2^

^1^ At the time of initial psychology consultation. ^2^ Determined by Medical Intensive Care Unit staff. ^3^ Confusion Assessment Method—Intensive Care Unit. ^4^ The Visual Analog Scale-Anxiety (VAS-A) ranges from “not anxious at all” (score = 0) to “most anxious I have ever felt” (score = 100). Note: ARF = Acute Respiratory Failure; RASS = Richmond Agitation-Sedation Scale.

**Table 2:** Self-Management for Acute Respiratory Failure (SMARF) Intervention.

SMARF Session	Session Description
1. Education & Goal Setting	Learning the relationship between medical events and changes in anxiety level; education about structure and format of sessions; set goals for SMARF treatment
2. Elicit Concerns & Problem Solving	Eliciting concerns about medical care (focusing on concerns within patient control); develop problem-solving skills & established approaches, including SMART (Specific, Measurable, Attainable, Relevant, Time-sensitive) goals
3. Thought Restructuring	Identifying the relationship between thoughts and anxiety, identifying & challenging maladaptive thoughts and beliefs
4. Relaxation training	Identifying the relation between anxiety and physiologic response, and acquiring relaxation skills (e.g., guided imagery, body scan, and breathing retraining)
5. Exposure	Understanding fear-avoidance patterns; providing in-vivo exposure to anxiety provoking events
6. Transition out of Hospital	Providing education about anxiety and discharge from hospital; identifying “red flags” about levels of anxiety; discussing how to find help in the next settings

**Table 3:** Characteristics of 11 Patients Receiving Self-Management Intervention.

Patient Characteristic	Statistic
Age, Median (IQR) Years	48 (32, 69)
Female, N (%)	6 (55)
Race/Ethnicity, N (%)	
White	6 (55)
Black	4 (36)
Hispanic Ethnicity	1 (9)
ICU Admission Diagnosis Category, N (%)	
Acute Hypoxemia Respiratory Failure	8 (73)
Chronic Obstructive Pulmonary Disease	1 (9)
Sepsis	1 (9)
Gastrointestinal Bleed	1 (9)
ICU LOS, median (IQR) days	47 (24, 58) ^1^
Hospital LOS, median (IQR) days	51 (30, 121)
ICU LOS prior to start of intervention, median (IQR) days	19 (13, 21)
Hospital LOS prior to start of intervention, median (IQR) days	25 (14, 49)

^1^ Total days in ICU during hospital stay (i.e., includes any ICU re-admission). Note: IQR = Interquartile range; ICU = Intensive Care Unit; LOS = Length of stay.

**Table 4:** Self-Management Intervention Data.

Intervention Result	Statistic
Agree to participate in intervention, N (%)	11 (100)
Number of sessions completed per patient, median (IQR)	6 (3, 6)
Pre-intervention VAS-A ^1^ score, median (IQR)	70 (57, 75)
Intervention sessions completed in ICU, N (%)	42 (84%)
VAS-A ^1^ decrease from pre-intervention to final session for all patients, median (IQR)	44 (21, 48)
VAS-A ^1^ decrease from pre-intervention to final session for patients receiving all 6 sessions, median (IQR)	51 (44, 62)

^1^ The Visual Analog Scale-Anxiety (VAS-A) ranges from “not anxious at all” (score = 0) to “most anxious I have ever felt” (score = 100). Note: IQR—Interquartile range.
